# A classification of idiopathic epiretinal membrane based on foveal avascular zone area using optical coherence tomography angiography

**DOI:** 10.1080/07853890.2024.2316008

**Published:** 2024-03-19

**Authors:** Zhengxi Zhang, Jianbo Mao, Jimeng Lao, Xinyi Deng, Yuyan Fang, Nuo Chen, Chenyi Liu, Yiqi Chen, Lijun Shen

**Affiliations:** a School of Ophthalmology and Optometry, Wenzhou Medical University, Wenzhou, China; b Department of Ophthalmology Center, Zhejiang Provincial People’s Hospital, Affiliated Hospital of Hangzhou Medical College, Hangzhou, China; c Affiliated Eye Hospital of Wenzhou Medical University, Hangzhou, China; d Yongkang Hospital, Yongkang, China; e Chicago College of Optometry, Midwestern University, Downers Grove, IL, USA

**Keywords:** Idiopathic macular epiretinal membrane, iERM, foveal avascular zone, pars plana vitrectomy, optical coherence tomography, optical coherence tomography angiography

## Abstract

**Objective:**

To evaluate the characteristics and prognoses of idiopathic macular epiretinal membrane (iERM) using a classification based on the foveal avascular zone (FAZ) area.

**Method:**

IERMs were classified into four stages based on the FAZ area. Baseline FAZ-related parameters, pre-and postoperative central macular thickness (CMT), and best corrected visual acuity (BCVA) were observed and compared between different stages. The correlations of structural parameters with pre-and postoperative logMAR BCVA were analyzed.

**Results:**

162 iERM eyes were enrolled, including 105 eyes followed up for 12 months after surgery. The preoperative BCVA was better at the early stage. Postoperative BCVA at Stages 2 and 3 were better compared to Stage 4. The early stage was associated with thinner CMT pre-and postoperatively. However, there was no significant difference in CMT between postoperative Stages 1 and 2 or Stages 3 and 4. Preoperative logMAR BCVA was negatively correlated with FAZ area, perimeter, and FD-300 and was positively correlated with CMT and acircularity index (AI). CMT correlated positively with BCVA for each stage, except Stage 4; FAZ area, perimeter, and FD-300 had a negative correlation at Stage 1. Baseline BCVA and CMT positively correlated with BCVA at the last follow-up, while FAZ area and FD-300 were negatively correlated. Baseline BCVA had a positive correlation for each stage, except Stage 1; FD-300 had a negative correlation at Stages 2 and 3; CMT had a positive correlation at Stage 3.

**Conclusion:**

A classification based on the FAZ area was established innovatively. This classification can reflect the progression of iERM and is helpful to the postoperative prognosis.

## KEY MESSAGES

(1) Classification based on FAZ area facilitate automation and consistency compared to the previous OCT-based qualitative grading.(2) With baseline FAZ stage advanced, thickened CMT and worsened BCVA was observed at baseline and 1-year post-operation. (3) Baseline FAZ area and FD-300 negatively correlated with logMAR BCVA at the last follow-up, reflecting the nonnegligible prognostic impact of macular vascular changes.

## Introduction

Idiopathic macular epiretinal membrane (iERM) is a common macular disorder without any identified causes. It grows on the inner limiting membrane’s surface, often distorting the retina’s integrality. Patients with iERM may complain about the loss of visual acuity, and metamorphopsia, aniseikonia symptoms [1,2]. Pars plana vitrectomy (PPV) with epiretinal membrane peeling is considered a safe and effective therapy for patients. However, sometimes fails to achieve sufficient anatomical and functional recovery after therapy [3–5].

Optical coherence tomography (OCT) is widely used to monitor and diagnose the disease of ERM. Many classification schemes of iERMs were established based on OCT. Govetto et al. [6] classified iERMs into four groups based on the shape of the central foveal and ectopic inner foveal layers (EIFL). Zur et al. [7] graded iERMs into three stages by the severity of the disorganization of inner retinal layers. However, there is no universal system to evaluate the severity of the disease using OCT-based systems [6–10]. Most of these grading schemes need human qualitative identification and are challenging to achieve automation, and often suffer from intra and inter-observer discrepancies. Recently, optical coherence tomography angiography (OCTA) has been used to observe the vessel changes of the macular regions, which may reflect the centripetal contractive forces of iERM that are not adequately imaged by structural OCT [8,11–13]. OCTA can provide an areal and morphological evaluation of the foveal avascular zone (FAZ), a region without retinal blood vessels at the central fovea. FAZ area-based grading, taking advantage of the automatic measurement of OCTA, can achieve automated grading and facilitate consistency. To the extent of our knowledge, such a proposal has not yet been presented in the related literature. A previous study found that the FAZ area in iERM eyes was reduced compared with normal eyes, and was related to preoperative logMAR BCVA in iERM eyes [14], as others reported [15,16]. However, studies on the correlation between the baseline FAZ area and postoperative visual function have been controversial and lacked long-term follow-up [15–21].

This study investigated 12-month surgical outcomes based on the FAZ area classification in patients with ERM. Additionally, we evaluated the feasibility and effectiveness of this classification based on FAZ in predicting the therapy response after therapy.

## Material and methods

### Study Participants

Participants diagnosed with iERM from November 2018 to February 2020 at Wenzhou Medical University Affiliated Eye Hospital were reviewed in this retrospective study. Inclusion criteria were (1) unilateral idiopathic epiretinal membrane; (2) aged ≥50 years; (3) all eyes with surgery underwent the 23-gauge standard 3-port PPV that included peeling of the ERM and internal limiting membrane (ILM) and combined cataract surgery. Exclusion criteria were (1) any other active ocular diseases such as ocular infection; (2) any other retinal or choroidal disease such as retinal detachment, age-related macular degeneration, diabetic retinopathy, hypertension retinopathy, glaucoma, or retinal vein occlusion; (3) any history of vitreoretinal surgery or ocular trauma; (4) any epiretinal membrane secondary to other retinal diseases; (5) eyes with an axial length ≥26 mm or refractive error greater than −6 dioptres (D); or (6) media opacities that prevented good visualization of the fundus. The health fellow eyes of these patients served as normal controls. Regional Ethics Committee approval of the study was obtained (the ethics approval number: 2019168K160).

The same experienced specialist (LS) completed all surgeries. Patients with posterior capsular opacity at follow-up will be treated before the examination. A postoperative follow-up period of fewer than twelve months was excluded from the postoperative analysis.

### Examination

All eyes underwent OCTA (Optovue RTVue XR Avanti; Optovue Inc., CA, USA; version 2017.1) imaging at the baseline visit. Images (3 × 3 mm) taken were centered on the fovea and included in the signal strength index analysis was ≥6. FAZ was defined as the central fovea region devoid of retinal blood vessels. The border of FAZ was automatically drawn with the Optovue software, whose reliability is acceptable [14,20,22,23]. Referring to the method Feng et al. [23] described, two experienced masked researchers (ZZ and JM) evaluated the accuracy of automated segmentation and performed manual adjustments if necessary. An expert’s (LS) opinion resolved discrepancies. FAZ-related parameters, described below, were then automatically measured on a retina slab, which was set to evaluate the region from the ILM to 10 µm below the outer plexiform layer.

As per routine clinical practice, central macular thickness (CMT) was evaluated by OCT (Heidelberg Spectralis OCT, Heidelberg Engineering, Heidelberg, Germany), and best-corrected visual acuity (BCVA) was taken at baseline, and the 1-, 3-, 6-, 12-months following surgery. BCVA was measured with the Chinese standard logarithm visual chart and then converted to a logMAR scale.

The primary outcome measures were BCVA, CMT, and the FAZ parameters of (1) area, (2) perimeter, (3) acircularity index (AI), determined as the ratio of the measured perimeter to a regular circle perimeter with the same FAZ area, and (4) the FD-300, determined as the foveal vessel density in a 300-μm wide region around the FAZ.

### Grading

**Figure 1.**
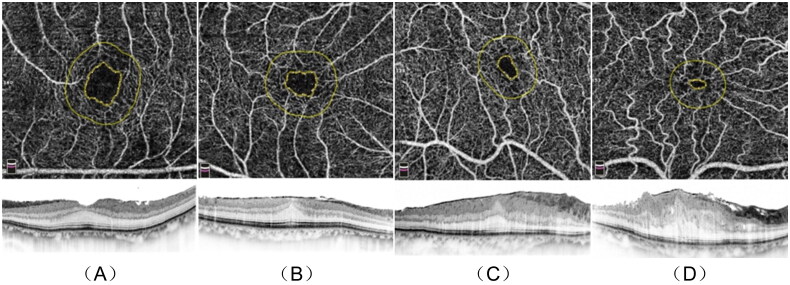
Stages 1 – 4 of iERM. *En-face* images (upper row). B-scan images (lower row) (A) Stage 1, FAZ area 0.23 mm^2^ (upper), CMT 296 μm (lower). (B) Stage 2, FAZ area 0.12 mm^2^ (upper), CMT 386 μm (lower). (C) Stage 3, FAZ area 0.06 mm^2^ (upper), CMT 572 μm (lower). (D) Stage 4, FAZ area 0.02 mm^2^ (upper), CMT 715 μm (lower). iERM: idiopathic macular epiretinal membrane; CMT: central macular thickness. (All OCTA scans were acquired in a 3x3mm setting.).

After excluding the ERM diagnosis and screening according to the above exclusion criteria, the average FAZ area of 143 healthy fellow eyes was 0.32 ± 0.12 mm^2^, which was similar to the previously reported values [8,16,18,24,25]. Therefore, affected eyes were graded into the following four stages (Figure 1): Stage 1, FAZ area ≥0.16 mm^2^ (≥50% area); Stage 2, 0.16 mm^2^ >FAZ area ≥0.08 mm^2^ (25% ∼ 50% area); Stage 3, 0.08 mm^2^ >FAZ area ≥0.04 mm^2^ (12.5% ∼ 25% area); Stage 4, FAZ area <0.04 mm^2^ (<12.5% area).

### Statistical analysis

All statistical analyses were performed using SPSS version 23.0 (SPSS, Chicago, IL, USA). Data were expressed as means ± standard deviations. One-way ANOVA (LSD test) was used to compare multiple groups. The paired-sample *t*-test was used for comparisons of preoperative and postoperative measurements. The chi-square or Fisher’s exact test was used to compare categorical variables. Correlation factors of logMAR BCVA were analyzed by Pearson correlation analysis. P-values <0.05 were considered statistically significant.

## Results

### Baseline characteristics

One eye from every 162 patients (age 64.3 ± 7.7 years; 28.4% male; 71.6% female) met the inclusion criteria. There were 40 eyes (24.7%) in Stage 1, 38 eyes (23.5%) in Stage 2, 44 eyes (27.2%) in Stage 3, and 40 eyes (24.7%) in Stage 4. There were no significant differences in patient age (*p* = 0.64) or sex ratio (*p* = 0.11) among the groups.

Forty-nine eyes were not performed surgical intervention because of mild symptoms or refused surgery, and eight did not complete the follow-up evaluations. Instead, they were enrolled only for baseline analyses. One hundred and five eyes (26.7% male, 73.3% female) completed surgery without any postoperative complication that contributed to the visual outcome, of which 5 (4.8%) were in Stage 1, 26 (24.8%) in Stage 2, 41 (39.0%) in Stage 3, and 33 (31.4%) in Stage 4.

### Preoperative measurements

Baseline BCVA was better at earlier iERM stages and poorer at advanced stages (*p* < 0.001, Table 1). Significant decreases were in the FAZ area and perimeter with more advanced stages and increases in CMT (all *p* < 0.001). Pairwise comparisons among all stages present statistical differences (all *p* < 0.05). The groups did not differ in AI (*p* = 0.20) or FD-300 (*p* = 0.27).

**Table 1.** Baseline measurements with FAZ area classification.

	Stage 1	Stage 2	Stage 3	Stage 4	*F*	*p*
BCVA (logMAR)	0.13 ± 0.15	0.29 ± 0.26	0.46 ± 0.29	0.63 ± 0.29	29.44	< 0.001
CMT (μm)	276.4 ± 56.9	407.4 ± 80.9	522.3 ± 112.5	579.9 ± 116.1	76.37	< 0.001
FAZ parameters						
FAZ area (mm^2^)	0.24 ± 0.04	0.11 ± 0.02	0.06 ± 0.01	0.02 ± 0.01	584.20	< 0.001
PER (mm)	2.06 ± 0.35	1.40 ± 0.20	1.01 ± 0.15	0.65 ± 0.18	21.34	< 0.001
AI	1.19 ± 0.17	1.19 ± 0.09	1.25 ± 0.29	1.29 ± 0.28	1.58	0.20
FD-300 (%)	47.43 ± 4.66	46.05 ± 5.80	45.71 ± 4.85	45.00 ± 6.93	1.32	0.27

FAZ: foveal avascular zone; BCVA: best-corrected visual acuity; logMAR: log (minimum angle of resolution); CMT: central macular thickness; PER: FAZ perimeter; AI: acircularity index; FD-300: the foveal vessel density in a 300-μm-wide region around the FAZ.

One-way ANOVA tests were used in all statistical analyses.

### Postoperative parameters

**Figure 2.**
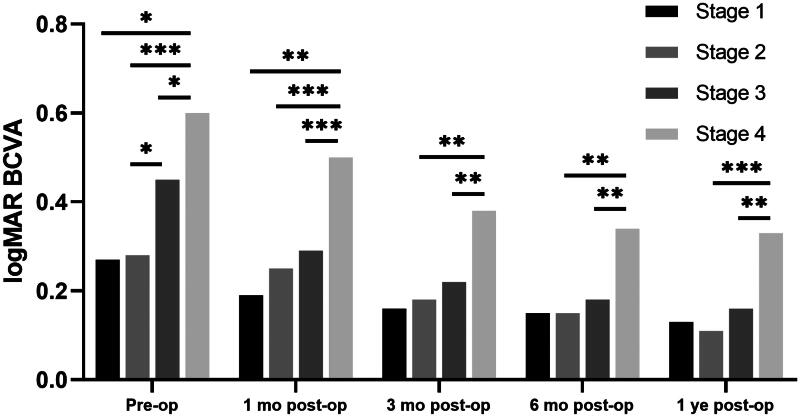
LogMAR BCVA in pre-  and postoperative iERM eyes with surgery. The significant differences in pairwise comparison were signed by “*”: * *p* < 0.05; ** *p* < 0.01; *** *p* < 0.001. BCVA: best-corrected visual acuity; logMAR: log (minimum angle of resolution); Pre-op: pre-operation; 1  month post-op: 1 month post-operation; 3  months post-op: 3 months post-operation; 6  months post-op: 6 months post-operation; 1 yearar post-op: 1 year post-operation. Independent sample *t*-tests were used in all statistical analyses.

There was a statistically significant improvement in BCVA at 12 months of follow-up compared to the baseline (0.44 ± 0.30 to 0.20 ± 0.25 logMAR, *p* < 0.001). At each postoperative visit, the BCVA of the earlier stages was better than that of the following stage (*p* < 0.005, Table 2). In pairwise comparisons (Figure 2), the BCVA was inversely related to the iERM stage, i.e.,, Stage 2 < Stage 4, Stage 3 < Stage 4 (*p* < 0.05). The BCVA difference between Stage 1 and Stage 4 was significant at 1-month post and marginallyly significant (*p* < 0.1) at other follow-up times.

**Table 2.** LogMAR BCVA in pre-  and postoperative iERM eyes with surgery.

	Pre-op	1 month post-op	3 months post-op	6 months post-op	1 year follow-up
Stage 1	0.27 ± 0.16	0.19 ± 0.09	0.16 ± 0.10	0.15 ± 0.11	0.13 ± 0.09
Stage 2	0.28 ± 0.20	0.25 ± 0.18	0.18 ± 0.18	0.15 ± 0.17	0.11 ± 0.16
Stage 3	0.45 ± 0.29	0.29 ± 0.21	0.22 ± 0.20	0.18 ± 0.17	0.16 ± 0.17
Stage 4	0.60 ± 0.31	0.50 ± 0.30	0.38 ± 0.29	0.34 ± 0.31	0.33 ± 0.34
F	6.44	7,71	4.79	4.90	5.68
P	<0.001	<0.001	0.004	0.003	0.001

BCVA: best-corrected visual acuity; log (minimum angle of resolution); Pre-op: pre-operation; post-op: post-operation; iERM: idiopathic macular epiretinal membrane.

One-way ANOVA tests were used in all statistical analyses.

**Figure 3.**
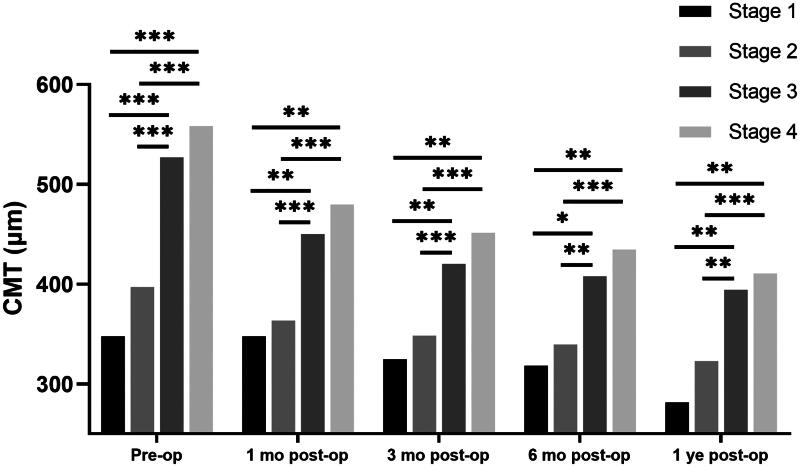
CMT in pre-  and postoperative iERM eyes with surgery. The significant differences in pairwise comparison were signed by “*”: * *p* < 0.05; ** *p* < 0.01; *** *p* < 0.001. CMT: central macular thickness; Pre-op: pre-operation; 1  month post-op: 1 month post-operation; 3  months post-op: 3 months post-operation; 6  months post-op: 6 months post-operation; 1 year post-op: 1 year post-operation. Independent sample t-tests were used in all statistical analyses.

There was a significant improvement in CMT 12 months after surgery (495.6 ± 120.4 to 375.9 ± 93.0 μm, *p* < 0.001). At each follow-up visit, the CMT of the earlier stages was thinner than that of the later stages (*p* < 0.001, Table 3). Pairwise comparisons showed that the CMT was inversely related to the iERM stage (Figure 3), i.e.,, the CMT at Stages 1 and 2 was thinner than that at Stages 3 and 4 (*p* < 0.05).

**Table 3.** CMT (μm) in pre- and postoperative iERM eyes with surgery.

	Pre-op	1 month post-op	3 months post-op	6 months post-op	1 year follow-up
Stage 1	347.8 ± 93.6	347.6 ± 96.4	324.8 ± 77.1	318.2 ± 75.0	281.4 ± 69.5
Stage 2	397.0 ± 62.0	363.5 ± 69.9	348.5 ± 65.0	339.6 ± 66.9	323.1 ± 62.0
Stage 3	527.0 ± 112.2	450.1 ± 82.1	420.3 ± 80.3	407.9 ± 84.7	394.4 ± 87.8
Stage 4	558.3 ± 105.5	479.8 ± 76.7	451.5 ± 75.8	434.7 ± 88.7	410.6 ± 99.4
F	18.00	13.39	11.45	8.29	7.69
P	<0.001	<0.001	<0.001	< 0.001	< 0.001

CMT: central macular thickness; iERM: idiopathic macular epiretinal membrane; pre-op: pre-operation; post-op: post-operation.

One-way ANOVA tests were used in all statistical analyses.

### Correlation analysis of BCVA pre-  and postoperatively

Generally, the preoperative FAZ area, FAZ perimeter, and FD-300 were negatively correlated with the baseline BCVA (Pearson correlation analysis, Table 4). In contrast, CMT and AI were positively correlated with it. When analyzed in different stages separately, CMT was positively correlated with BCVA at Stages 1–3. FAZ area, FAZ perimeter, and FD-300 are are only negatively correlated with BCVA at Stage 1.

**Table 4.** Correlation analysis of preoperative logMAR BCVA.

	All		Stage 1		Stage 2		Stage 3		Stage 4	
	*r*	*P*	*r*	*P*	*r*	*P*	*r*	*P*	*r*	*p*
CMT	0.70	<0.001	0.56	<0.001	0.74	<0.001	0.51	<0.001	0.29	0.076
FAZ area	−0.61	<0.001	−0.72	<0.001	−0.17	0.314	−0.13	0.393	−0.13	0.422
PER	−0.39	<0.001	−0.42	0.007	0.12	0.488	0.01	0.976	−0.18	0.274
AI	0.16	0.04	−0.05	0.780	0.32	0.051	−0.09	0.550	0.27	0.095
FD-300	−0.20	0.012	−0.37	0.018	−0.16	0.347	−0.26	0.093	0.05	0.754

BCVA: best-corrected visual acuity; log (minimum angle of resolution); CMT: central macular thickness; FAZ: foveal avascular zone; PER: FAZ perimeter; AI: acircularity index; FD-300: the foveal vessel density in a 300-μm-wide region around the FAZ.

Pearson correlations were used in all statistical analyses.

Correlation analyses were performed to determine if the baseline variables were correlated with a a better final BCVA (Table 5). Overall, baseline BCVA and CMT were positively correlated with the final BCVA, while FAZ area and FD-300 were negatively correlated factors. When analyzed in different stages separately, the baseline BCVA was positively correlated with the final BCVA at Stages 2–4. CMT was positively correlated with the final BCVA only at Stage 3, while FD-300 was negatively correlated at Stages 2 and 3.

**Table 5.** Correlation analysis of 1-year follow-up logMAR BCVA in eyes with surgery.

	All		Stage 1		Stage 2		Stage 3		Stage 4	
	*r*	*P*	*r*	*P*	*r*	*P*	*r*	*P*	*r*	*P*
Baseline BCVA	0.52	<0.001	−0.12	0.851	0.58	0.002	0.39	0.012	0.49	0.004
CMT	0.39	<0.001	0.49	0.402	0.33	0.099	0.44	0.004	0.24	0.191
FAZ area	−0.30	0.002	0.04	0.946	−0.11	0.586	−0.05	0.74	−0.07	0.695
PER	−0.18	0.086	0.15	0.806	−0.04	0.865	−0.15	0.366	−0.15	0.447
AI	0.18	0.067	0.11	0.857	0.18	0.381	−0.16	0.304	0.27	0.135
FD-300	−0.24	0.021	−0.18	0.777	−0.54	0.005	−0.41	0.009	−0.10	0.623

BCVA: best-corrected visual acuity; log (minimum angle of resolution); CMT: central macular thickness; FAZ: foveal avascular zone; PER: FAZ perimeter; AI: acircularity index; FD-300: the foveal vessel density in a 300-μm-wide region around the FAZ.

Pearson correlations were used in all statistical analyses.

## Discussion

In contrast to previous iERM classifications based mainly on OCT qualitative measurements, we proposed a classification scheme using quantitative OCTA measurements. The baseline FAZ area and perimeter were reduced with each higher iERM stage, while the CMT was increased, and each was correlated with the worse baseline BCVA. The advanced stage was associated with a prognosis of thicker CMT and worse BCVA. Baseline FAZ area, CMT, and FD-300 were correlates of their 1-year postoperative BCVA for eyes undergoing surgery. Thus, the iERM stage based on the FAZ area could benefit preoperative assessment and postoperative estimation.

IERM exposes the macula to anteroposterior and tangential stresses, resulting in macula thickening and vascular shift [14,26]. Previous studies identified OCT predictors for structural and functional outcomes after surgery, such as the thickness of the inner nuclear layer and CMT and defects of the interdigitation zone and ellipsoid zone [27–30]. The FAZ area is automatically estimated by the software of the OCTA system and included in the report, making it simpler to quantify than other characteristics, such as inner nuclear layer thickness.

Previous histopathological studies of iERM tissue specimens found centripetal thickening of fibrous structures [31]. In this research, preoperative BCVA was worse, and CMT thickened with stage progression, reflecting the synchronous actions of the anteroposterior and tangential forces of the iERM. Similarly, Ersoz et al. observed a correlation between FAZ area and central foveal thickness [20], and Shiihara et al. [32] reported the perimeter decreased with disease progression. Although AI increased and FD-300 decreased with the advanced stage, none of the differences were statistically significant. The previous investigation found lower FD-300 in afflicted eyes than in controls, and AI was marginally different [14]. Other influencing factors may cause AI and FD-300 not to vary regularly with decreasing FAZ area. BAE et al. [5] proposed the A-zone to represent the area of complete attachment between ERM and ILM. They reported that the A-zone and retinal folds involved the central foveal in some affected eyes and correlated with M-score, which may also affect FD-300 and AI.

Overall correlation analyses of baseline BCVA showed that CMT and AI were positive, while FAZ area, FAZ perimeter, and FD-300 were negative. The correlation between FAZ area and baseline BCVA was also proven in previous studies [8,14–16,21]. When discussed solely in groups, the thicker the macula, the worse the visual acuity in Stages 1, 2, and 3. Since stage 4 iERMs are an exception, we hypothesize that some microstructural damage might have produced major functional changes: mechanical stretching of the retinal pigment epithelium may increase levels of vascular endothelial growth factor, leading to increased retinal and choroidal vascular permeability [33,34] correlates with BCVA [35]. Microcystoid macular oedema (MME) is linked to advanced iERM, affects BCVA [36], and may be caused by Müller cell loss or malfunction [37] or retrograde trans-synaptic bipolar cell degeneration secondary to retinal nerve fiber traction [38]. Visual correlations of baseline BCVA with FAZ area, FAZ perimeter, and FD-300 were only found at Stage 1, showing that the FAZ parameters were related to early visual loss. There is controversy over whether such a correlation exists between FAZ parameters and baseline BCVA [14,19,20,32] might be because of the limited sample size.

BCVA improved, and CMT became thinner within the 12-month follow-up period. Eyes with earlier stages at baseline presented better BCVA and thinner CMT in each follow-up period. The retracted foveal vascular system may scatter light before it reaches the photoreceptor cells [26], and centripetal displacement of the end feet of Müller cells leads to probable abnormal light guiding [39–41]. Even 1 year after surgery, differences in BCVA between preoperative stages persisted. In the post hoc multiple comparison tests, the analysis of Stage 1 was limited because of the inadequate sample size with surgery (5 cases). Of note is that there was a significant difference in BCVA between stage 3 and stage 4 at each postoperative follow-up point, whereas there was no significant difference in CMT. One potential explanation is that Stage 4 eyes had microstructural damage in addition to the simple thickening of the CMT, which makes it harder to recover. As discussed above, MME, which is more likely to occur in advanced iERM, is associated with poor postoperative visual recovery and less efficient inflammation control [36].

Similar to our results, previous research reported that BCVA recovery was negatively related to the FAZ area [8,15,17,22] for postoperative correlation analyses. However, several studies reported controversial results that may be attributed to the varying sample sizes, instruments, and measurement depth [14,16,18,20]. CMT was positively correlated with the last follow-up BCVA in totality and at Stage 3. However, it was absent at Stage 4, possibly because of the microstructural damage that likely exists at Stage 4. The FD-300 was positively related for all and iERM Stages 2 and 3. Previous studies on FD-300 reported that the recovery at six months postoperatively did not reach statistical significance. The absence of a correlation with postoperative visual acuity was probably attributed to the limited sample size and insufficient follow-up period [8, 20]. It can be hypothesized that the vascular density changes around the FAZ, restored slowly after surgery, could be associated with long-term effects on postoperative visual outcomes.

OCTA helps study the natural history and postoperative progression of vitreoretinal lesions from the standpoint of each vascular layer plane. However, there are still discrepancies across devices because of varying angiographic algorithms and measurement ranges. Previous investigations [42–45] have validated the variations in FAZ measurements. Thus, we showed a quantitative grading system based on FAZ size: although FAZ detected discrepancies across devices, most studies indicate minor numerical differences [43–46]. The viability of a quantitative grading technique to lessen the influence of device variations and improve the generalizability of the findings will be examined in the future. The measured values between different devices will likely get closer as hardware and software accuracy improves. Nevertheless, until then, this quantitative grading technique may be a means to mitigate the effect of device variances.

The limitations of this study include (1) the retrospective nature and (2) the small sample size, especially since most Stage 1 patients had mild symptoms and were not treated surgically. The possibility of disease progression in non-surgical patients during long-term follow-up is also worth exploring. (3) This study evaluated only BCVA as a marker of visual function. Metamorphopsia and aniseikonia are common symptoms that warrant further research to determine if the the FAZ classification is correlated with these indexes. (4) This study aims to test a quantitative grading method based on OCTA. However, there are variances in measurement outcomes across various devices, and future research is warranted to evaluate if it is widely applicable. OCTA-related measures like the FAZ area may be affected by artefacts and diurnal fluctuations; this study failed to control the examination time due to its retrospective nature. Large-scale, well-controlled, prospective studies are required in the future. In summary, this study established an innovative classification method based on the FAZ area, revealing the progression of iERM disease by decreasing the FAZ area. The change in FAZ is an early indicator of visual effects. Postoperative analysis showed that the prognosis of BCVA was poor when the disease progressed to Stage 4. Although, based on the study results, there was still a significant difference in the final follow-up CMT between Stage 2 and 3, patients with iERM equal to or greater than Stage 2 were more likely to require surgical intervention for a better prognosis.

## Data Availability

The data supporting this study’s findings are available on reasonable request from the corresponding author, Shen L. However, the data are not publicly available because they contain information that could compromise the privacy of the research participants.
